# Physical exercise and subjective wellbeing among Chinese students in South Korea: the mediating roles of exercise-related psychological needs satisfaction and psychological resilience

**DOI:** 10.3389/fpsyg.2026.1817629

**Published:** 2026-06-26

**Authors:** Shang Gao, Mingjin Yan, Xinxin Zhang, Wei Sun

**Affiliations:** 1 Myongji University - Natural Science Campus, Yongin-si, Republic of Korea; 2 Zhengzhou University, Zhengzhou, China

**Keywords:** Chinese international students, physical exercise, psychological needs satisfaction, psychological resilience, subjective wellbeing

## Abstract

As the number of Chinese students studying in South Korea continues to increase, understanding factors associated with their subjective wellbeing (SWB) has become increasingly important. Physical exercise is widely recognized as beneficial for mental health; however, its underlying mechanisms remain underexplored. Drawing on Self-Determination Theory and resilience theory, this study examines the association between physical exercise and SWB, as well as the mediating roles of exercise-related psychological needs satisfaction and psychological resilience. A total of 435 Chinese students from six universities in South Korea participated in the study. Data were collected using the Physical Activity Rating Scale (PARS-3), Basic Psychological Needs in Exercise Scale (BPNS-EX), Connor–Davidson Resilience Scale (CD-RISC), and International Wellbeing Scale (IWB). Correlation analysis and chain mediation analysis were conducted using SPSS 26.0 and PROCESS macro. Results showed that physical exercise was positively associated with SWB (*β* = 0.230, *p* < 0.001). This association was partially mediated by psychological needs satisfaction (*β* = 0.038), psychological resilience (*β* = 0.015), and their sequential pathway (*β* = 0.005), with the total indirect effect accounting for 25.2% of the total effect. These findings suggest that physical exercise is associated with improved wellbeing both directly and indirectly through psychological mechanisms. The results provide implications for promoting international students’ psychological adjustment through structured physical activity.

## Introduction

Over the last few years, as a result of the ever-increasing globalization of the Chinese higher education system, the international in-country students have continued to expand. Official statistics published by the Ministry of Education show that the total number of Chinese students studying abroad exceeded 700,000 as of 2022. Along with other places, South Korea is a leading destination for Chinese students due to geographical proximity and cultural similarity (Ministry of Education, 2020). Nonetheless, cross-cultural adaptation is usually characterized by difficulties, which include academic pressure, cultural shock as well as social isolation among international students. These challenges have been associated with negative mental health outcomes and lower subjective wellbeing (SWB; Brunsting et al., 2018; Smith and Khawaja, 2011; Wilczewski and Alon, 2023).

It is on this context that the Chinese government as well as the South Korean educational institutions have implemented various policy measures that seek to promote mental adaptation and livelihood of the international students (State Council, 2016; Ministry of Culture, Sports and Tourism, Republic of Korea, 2022). An example of it is the fact that the Ministry of Education of China has specifically focused on improving the mental health of international students in its “Study in China Programme” and promotes the latter by engaging in physical exercise, cultural exchange and similar activities (Ministry of Education, 2020). Equally, it is possible to note that in the context of its Support Plan to Foreign Students, the Ministry of Education of South Korea mentions its focus on facilitating the physical and mental wellbeing of international students by increasing their access to sports facilities on campus and offering mental health services (Ministry of Education, Republic of Korea, 2021).

### The relationship between physical exercise and subjective wellbeing among Chinese students in South Korea

Subjective wellbeing (SWB), which refers to a personal emotional and cognitive judgment of a person regarding his or her quality of life (Hascher and Waber, 2021; Van Agteren et al., 2021), has turned into a crucial indicator of the psychological wellbeing of international students. The Chinese students in South Korea mostly face a problem of low rates of subjective wellbeing as a result of the cultural adaptation problems, academic pressure, and lack of social support (Zhang and Goodson, 2011). Physical exercise has achieved popularity within recent years as a non-drug-based intervention method that is effective in improving mental health (De Nys et al., 2022; Penedo and Dahn, 2005). Nevertheless, how exactly the physical exercise affects the SWB of Chinese education students in the South Korea is not well comprehended yet and is subject to additional empirical studies.

Available evidence demonstrates that exercise enhances subjective wellbeing in a complex of several psychologically interconnected mechanisms. First, physiologically, consistent exercise activates the production of such neurotransmitters like endorphins, serotonin, and dopamine, thus relieving anxiety and depression symptoms (Basso and Suzuki, 2017; Chen and Nakagawa, 2023; Mandolesi et al., 2018). Second, socially-psychologically speaking, exercise reinforcements social bonds, meaning that with the help of it, international students develop new social networks and alleviate loneliness (Eather et al., 2023; Jackson et al., 2021; Martín-Rodríguez et al., 2024). As an example, Zerengok et al. (2018) discovered that international students engaged in team sports (i.e., soccer and basketball) had a greater level of life satisfaction and positive affect than non-exercisers. Moreover, physical exercise improves self-efficacy among individuals thus enabling them to be confident when dealing with the issue of cross-cultural adaptation (Bandura, 1997).

Nevertheless, the current studies on the topic of physical exercise and subjective wellbeing among international students are not comprehensive in multiple aspects (Lee et al., 2019; Hilber, 2024). On the one hand, the majority of the research has been carried out on the West, and the relevance of studying international student groups in Asian settings such as South Korea is not paid much attention (Heng, 2018; Kristiana et al., 2022). Because of cultural differences, educational systems, and social support structures of South Korea and China, the psychology of adaptation of Chinese students in South Korea may not be the same as it can be in the Western countries (Lee and Lee, 2020). Conversely, despite the positive effect of exercise on mental health, the mediating processes of exercise on mental health have not been effectively studied. As an illustration, psychological fulfillment (i.e., happiness and achievement one feels when engaging in physical exercise) and psychological resilience (i.e., a person being able to adapt to and to recover the stress) as exercise mechanisms could become the crucial mediating factors that work out on subjective wellbeing (Blustein et al., 2023; Khaldi et al., 2023).

### Physical exercise, psychological needs satisfaction, and subjective wellbeing

The relationship between physical exercise and subjective wellbeing has attracted considerable attention in positive psychology and exercise psychology. A growing body of research suggests that regular physical exercise is positively associated with higher levels of subjective wellbeing (Lin et al., 2022; Wang et al., 2024; Zhang et al., 2022). It is also possible, based on the Self-Determination Theory (SDT), that this positive effect is due to the place of exercise in fulfilling the basic psychological needs (Ryan and Deci, 2017). As people feel the satisfaction of the three general psychologic needs, namely autonomy, competence, and relatedness, at the time they exercise, they experience an increase of positive affect and life satisfaction (Bandhu et al., 2024; Göttgens and Oertelt-Prigione, 2021).

In the frame of the cross-cultural adaptation, physical exercise might be specifically important in terms of fostering subjective wellbeing. In their research on the use of physical exercise by Asian international students, Song et al. (2021) stated that moderate-intensity physical exercise among students three or more times per week had reduced the occurrence of depressive symptoms by 42% versus participants who did not do any physical exercise. Such protection was noticeable especially in the accomplishment of autonomy needs students were free to decide independently on the kind and intensity of exercise; then the stress on cross-cultural adaptation dramatically reduced (Yang, 2019). Interestingly, the sense of competence in the exercise showed the best predictive influence on wellbeing, which is most consistent with the culture of Chinese society which is characterized by the focus on the development of capabilities (Kim and Kuan, 2022; Yin et al., 2024).

The personality needs fulfillment in exercise is perhaps a primary mediating factor between physical exercise and subjective wellbeing. A longitudinal study by Ng et al. (2011) revealed that a 12-week Tai Chi intervention resulted in an increase of 27% of psychological needs satisfaction in the experimental group, which in turn resulted in a 19% increment in positive emotion scale on a subjective wellbeing scale. In group sports situations in particular, the relatedness need will contribute greatly to the alleviation of loneliness among the international student, and this social connection effect is stronger in individualistic cultural background (Kim and Park, 2021).

### Physical exercise, psychological resilience, and subjective wellbeing

The association of physical exercise and psychological resilience has emerged as one of the areas of concern in the fields of positive psychology and health psychology. According to a large number of studies, regular physical exercise notably improves psychological strength (Ávila et al., 2021; Cosco et al., 2017; Toth et al., 2023). The ability of an individual to be psychologically resilient, which can be defined as the ability to cope with stress and adversity, can be an important mediating variable between physical exercise and subjective wellbeing (Fletcher and Sarkar, 2016). The Stress Buffering Theory states that, physical exercise can also alleviate the negative impact of stress on the subjective wellbeing by increasing mental strength (Ong et al., 2010). Studies of international learners indicate that participants who experience moderate-intensity physical activities three or more times weekly indicate highly notable levels of psychological resilience in comparison to sedentary respondents, and such a relationship is particularly very strong among those who find themselves constantly facing an elevated degree of cross-cultural adaptation stress (Yim et al., 2023).

The psychological strengthening with physical exercise can take place through many different processes. According to neurobiological studies, one aspect of exercise that enhances emotional control is the secretion of brain-derived neurotrophic factor (BDNF), which is also found to strengthen the prefrontal executive functioning, and thus prefrontal cortex (Hillman et al., 2008). On the behavioral level, the process of challenge-response that come as an inherent part of physical exercise, develops the self-efficacy that is the primary element of psychological resilience (Bandura, 2018). Moreover, the process of goal setting and goal achieving inherent in exercise creates optimism, which is strongly considered to be one of the primary predictors of psychological strength (Seligman, 2006).

The present study adds to the existing literature by examining the relationship between physical exercise and subjective wellbeing among Chinese international students in South Korea, a group that has received comparatively limited attention in prior research, particularly in Asian contexts. By bringing together Self-Determination Theory and resilience theory, this study considers multiple psychological processes within a single analytical framework, allowing for a more nuanced understanding of how physical exercise is related to subjective wellbeing. In addition, the use of a chain mediation model makes it possible to explore how exercise-related psychological needs satisfaction and psychological resilience may operate in sequence, offering further insight into the potential pathways linking physical exercise and wellbeing.

From a practical standpoint, the findings may be relevant for universities and practitioners working with international student populations. In particular, the results suggest that participation in physical exercise could be considered as part of broader efforts to support students’ psychological adjustment and wellbeing during cross-cultural transitions.

### The chain-mediated effect of exercise-induced psychological need satisfaction and psychological resilience

This study integrates Self-Determination Theory (SDT) and resilience theory within a unified framework. According to SDT, physical exercise can satisfy individuals’ basic psychological needs for autonomy, competence, and relatedness, which are essential for psychological functioning (Ryan and Deci, 2017).

The satisfaction of these psychological needs may, in turn, enhance individuals’ psychological resilience by strengthening their sense of control, self-efficacy, and emotional regulation capacity. In this way, resilience can be understood as a downstream psychological resource that develops partly through need satisfaction.

This integrated perspective suggests a sequential process in which physical exercise first promotes psychological needs satisfaction, which then facilitates the development of psychological resilience, ultimately contributing to higher subjective wellbeing.

**Figure 1:**
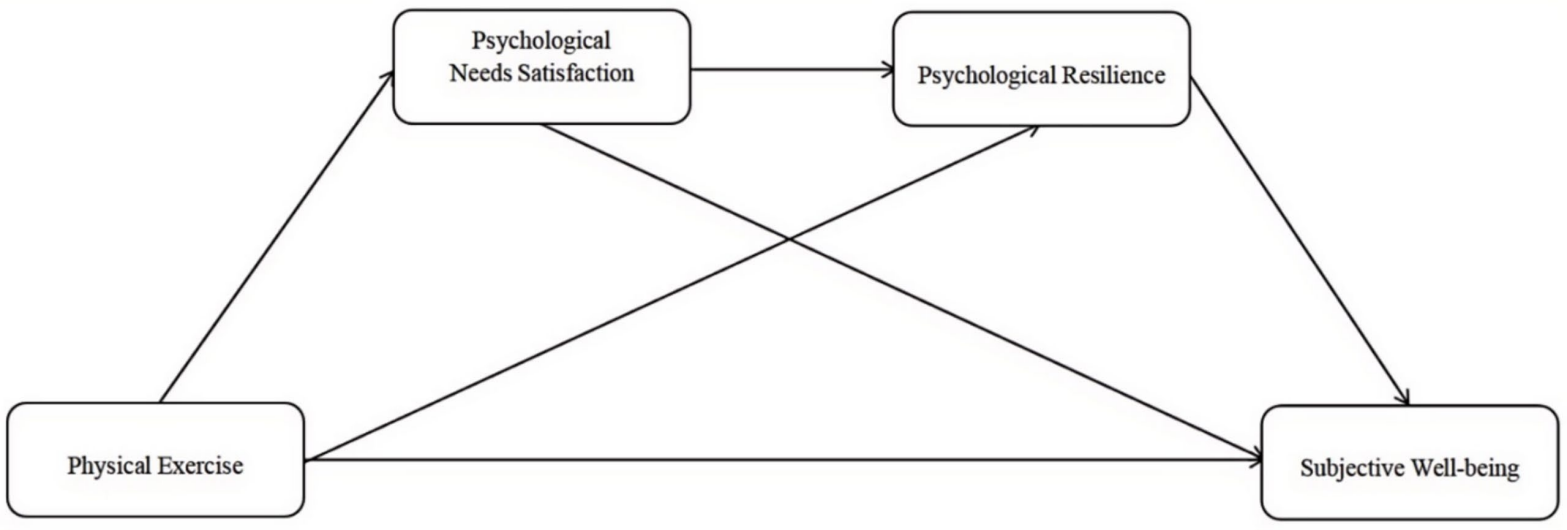
Hypothetical model diagram.

This study proposes the following hypotheses (see Figure 1):

H1: Physical exercise is positively associated with subjective wellbeing.

H2: Exercise-related psychological needs satisfaction is associated with the relationship between physical exercise and subjective wellbeing.

H3: Psychological resilience is associated with the relationship between physical exercise and subjective wellbeing.

H4: Exercise-related psychological needs satisfaction and psychological resilience may function as sequential mediators linking physical exercise and subjective wellbeing.

## Research methodology

### Research participants

The sampling used in this study was based on a convenience sampling approach to recruit Chinese international students studying at five major universities in South Korea (Seoul National University, Korea University, Yonsei University, Pusan National University, and Chungnam National University), located in Seoul, Busan, and Daejeon.

Participants were recruited through campus announcements, international student groups, and faculty recommendations, and participation was voluntary.

The following inclusion criteria were applied: (1) full-time undergraduate or graduate enrollment; (2) a minimum residence period of 6 months in South Korea; (3) no severe physical or mental health conditions; and (4) voluntary participation with written informed consent.

A total of 487 questionnaires were initially distributed, and 435 valid responses were retained after excluding incomplete or invalid questionnaires, resulting in an effective response rate of 89.3%.

The sample included 233 males (53.6%) and 202 females (46.4%), with ages ranging from 18 to 32 years (M = 25.3, SD = 4.4). The participants consisted of 127 undergraduates (29.2%), 223 master’s students (51.3%), and 85 doctoral students (19.5%). The duration of residence in South Korea ranged from 6 months to 5 years (M = 3.1, SD = 1.5).

The study was reviewed and approved by the Institutional Review Board of Binzhou University (BZUIRB-20250813). All data were anonymized and collected in accordance with the ethical principles of the Declaration of Helsinki.

### Research tools

#### Physical exercise rating scale

In this study, the Chinese version of the physical exercise Rating Scale (PARS-3), revised by Liang (1994), was used to assess the level of physical exercise among Chinese international students in South Korea. The scale has been widely used in studies involving Chinese populations and has demonstrated acceptable reliability and validity.

It consists of three dimensions—intensity, frequency, and duration—each rated on a 5-point scale (1 = light exercise; 5 = high-intensity sustained exercise). The total physical exercise score was calculated using the standard PARS-3 formula: exercise volume = intensity × frequency × (duration − 1), with higher scores indicating greater levels of physical exercise.

The Chinese version of the scale was used directly, as all participants were native Chinese speakers, and this version has been validated in prior studies involving Chinese university students.

Although this value is slightly below the commonly recommended threshold of 0.70 (Nunnally and Bernstein, 1994), it may still be considered acceptable given the brief structure of the scale and its limited number of items. In addition, the PARS-3 has been widely used in studies involving university student populations (Li et al., 2024).

Data were collected using a self-report method, and participants were asked to recall their physical exercise over the previous month to reduce potential recall bias.

#### Exercise psychological needs satisfaction scale

The psychological needs satisfaction scale (BPNS-EX) as revised by Zhu (2014) was used in this study. The scale is based on Self-Determination Theory (SDT) and it can measure psychological needs satisfaction in the exercise contexts under three dimensions, i.e., competence, autonomy and relatedness. The tool is made of 18 items (six of those per dimension), which are rated on a 6-point Likert scale between 1 (completely disagree) and 6 (completely agree). The increase in the overall scores will mean that there is more fulfillment of psychological needs in the course of exercise. To enhance the psychosomatic feelings of competence (e.g., I can successfully reach my exercise goals), autonomy (e.g., I choose exercise techniques I like), and relatedness (e.g., I feel happy to exercise with my peers) were introduced. This scale is common in the Chinese studies of sports psychology and has been shown to be construct valid and internally consistent. It has shown good psychometric qualities in this research (Cronbach’s *α* = 0.786, *x*^2^/df = 2.041, CFI = 0.962, TLI = 0.956, RMSEA = 0.049, SRMR = 0.030).

Given the focus of the current study, additional validity evidence was primarily based on CFA results and internal consistency estimates. Although convergent and discriminant validity were not examined separately, the satisfactory model fit and theoretical consistency of the three-factor structure provide support for the construct validity of the instrument in this context.

#### Psychological resilience scale

The Chinese version of the Connor-Davidson Resiliency Scale (CD-RISC) which was created by Yu and Zhang (2007) was used to measure psychological resilience. This tool is a 25-item assessment measure of three constructs, namely Tenacity (13 items), Strength (8 items), and Optimism (4 items). The items are evaluated on a 5-point Likert scale, with 0 (never) to 4 (always) being the possible answers, which come up with a total score of 0–100 with a high score signifying more psychological resilience. Tenacity indicates stamina and flexibility to stress (e.g., I can cope with setups); strength means the ability to overcome pressure when facing difficulties (e.g., I become stronger after facing troubles); and optimism is positive cognitive needs of the future (e.g., I believe things would always get better).

The 25-item version was selected because it provides a more comprehensive assessment of resilience across multiple dimensions, which is particularly relevant for understanding adaptation processes in cross-cultural contexts. The scale has proved to be very suitable to the Chinese cultural setting, as well as has been extensively tested in international student mental health studies. It showed good reliability and validity in this research (Cronbach’s *α* = 0.862, *x*^2^/df = 2.104, CFI = 0.913, TLI = 0.947, RMSEA = 0.050, SRMR = 0.028).

#### Index of wellbeing

The Index of WellBeing (IWB) by Campbell et al. (1976) was used to determine subjective wellbeing. This instrument is a 9-item measure, which is divided into two parts: the General Affective Index (including 8 items) and the life Satisfaction (including 1 item), with a 7-point Likert scale. Greater cumulative scores describe an improved subjective wellbeing. General Affective Index evaluates positive (e.g., I feel happy) and negative (e.g., I feel anxious, reverse-scored) as well as Life Satisfaction is evaluated by a single item of global assessment (e.g., I am satisfied with my current life). It has shown a high cross-culture applicability and is common in the studies involving the psychological adaptation of international students. It demonstrated a high reliability and validity in the current research (Cronbach’s *α* = 0.912, *x*^2^/df = 2.226, CFI = 0.983, TLI = 0.978, RMSEA = 0.053, SRMR = 0.026).

### Data statistics and analysis

Data were collected using a self-report questionnaire. The survey was administered primarily in an online format between [2025-09] and [2025-11]. Participants accessed the questionnaire through links distributed via student groups and campus communication channels.

To reduce potential social desirability bias, participants were informed that their responses would remain anonymous and confidential, and that there were no right or wrong answers. Participation was entirely voluntary, and respondents were encouraged to answer honestly based on their own experiences.

The study employed SPSS 26.0 for descriptive statistics, correlation analysis, and common method bias testing. The chained mediation model was validated using the SPSS macro Process Model 6.

## Results

### Common method Bias test

In an attempt to investigate possible common method bias, a single-factor test proposed by Harman was done. The items analyzed comprised 55 and eight factors with eigenvalues of over 1 were extracted. The former explained 14.127% of the total variance, which is significantly lower than the critical value of 50 which means that common method bias can hardly be considered a serious risk to the validity of the results. This cumulative variance found in the factors extracted amounted to 62.167 percent, which is more than the widely-used cutoff at 60 percent indicating that the factor structure was sufficiently able to explain the variance in the data.

### Correlation analysis

Table 1 presents the descriptive statistics and Pearson correlation coefficients among the study variables. Physical exercise was positively associated with exercise psychological needs satisfaction (*r* = 0.212, *p* < 0.01), psychological resilience (*r* = 0.186, *p* < 0.01), and subjective wellbeing (*r* = 0.234, *p* < 0.01).

**Table 1:** Descriptive statistics and correlations among study variables (*N* = 435).

Variable	M	SD	1	2	3	4
1. Physical exercise	28.218	34.417	–			
2. Exercise psychological needs satisfaction	3.434	0.914	0.212**	–		
3. Psychological resilience	2.005	0.768	0.186**	0.262**	–	
4. Subjective wellbeing	4.045	1.510	0.234**	0.249**	0.185**	–

Exercise psychological needs satisfaction was also positively related to psychological resilience (*r* = 0.262, *p* < 0.01) and subjective wellbeing (*r* = 0.249, *p* < 0.01). In addition, psychological resilience showed a positive association with subjective wellbeing (*r* = 0.185, *p* < 0.01).

Overall, the correlations among the variables were in the expected direction.

### Testing the chain mediation model

The proposed chain mediation model was tested with the help of the PROCESS macro (Model 6) in SPSS. The control variables were gender, age, education level and the duration of years study abroad. Psychological resilience and psychological needs satisfaction as the mediators of effects were both tested at the same time.

According to Table 2, physical exercise had a significant and positive impact on predicting subjective wellbeing (*β* = 0.230, *t* = 4.841, *p* < 0.001). The introduction of the mediating variables in the model demonstrated that physical exercise was a substantive predictor of exercise psychological needs satisfaction (*β* = 0.208, *t* = 4.379, *p* < 0.001) and psychological resilience (*β* = 0.139, *t* = 2.913, *p* < 0.01). Exercise psychological needs satisfaction also had a significant positive relationship with psychological resilience (*β* = 0.224, *t* = 4.719, *p* < 0.001) and subjective wellbeing (*β* = 0.181, *t* = 3.757). Subjective wellbeing was also largely predicted by psychological resilience (*β* = 0.106, *t* = 2.199, *p* < 0.05). Following the work of the mediating variables, the direct relatedness of physical exercise on subjective wellbeing was still significant (*β* = 0.172, *t* = 3.609, *p* < 0.001) which is partial mediation. The above findings indicate that psychological needs satisfaction and psychological resilience are the mediators of relationship between physical exercise and subjective wellbeing in part.

**Table 2:** Mediating role of exercise psychological needs satisfaction and psychological resilience in the relationship between physical exercise and subjective wellbeing.

Variable	Subjective wellbeing		Exercise psychological needs satisfaction		Psychological resilience		Subjective wellbeing	
	*β*	*t*	*β*	*t*	*β*	*t*	*β*	*t*
Physical exercise	0.230	4.841***	0.208	4.379***	0.139	2.913**	0.172	3.609***
Exercise psychological needs satisfaction					0.224	4.719***	0.181	3.757***
Psychological resilience							0.106	2.199*
Gender	−0.005	−0.053	−0.088	−0.934	−0.105	−1.137	0.024	0.262
Age	−0.014	−1.360	−0.016	−1.522	−0.001	−0.051	−0.011	−1.066
Education level	0.016	0.276	0.089	1.549	0.093	1.639	−0.012	−0.217
Duration of study abroad	−0.006	−0.184	−0.025	−0.756	−0.026	−0.817	0.002	0.057
*R*	0.243		0.241		0.31		0.33	
*R* ^2^	0.059		0.058		0.096		0.109	
*F*	5.391***		5.305***		7.592***		7.447***	

Among the control variables, gender, age, education level, and duration of study abroad did not demonstrate significant predictive effects. The explanatory power (*R*^2^) of the models ranged from 0.059 to 0.109, and all regression models were statistically significant (*p* < 0.001), indicating acceptable explanatory capacity.

The findings were consistent with the proposed model, indicating that physical exercise was associated with subjective wellbeing both directly and indirectly through exercise psychological needs satisfaction and psychological resilience.

**Figure 2:**
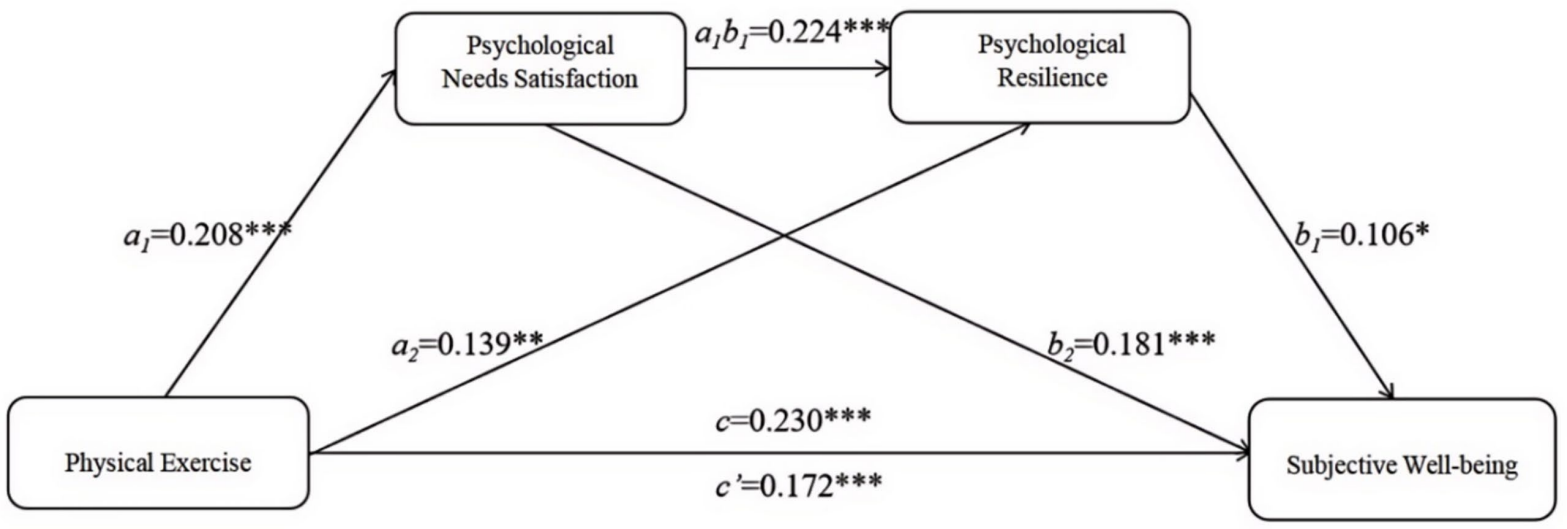
Chain mediation model of exercise-induced psychological need satisfaction and psychological resilience on physical exercise and subjective wellbeing.

Figure 2 and Table 3 present the results of the chain mediation analysis based on bootstrap sampling (5,000 resamples).

**Table 3:** Bootstrap tests for chain mediation effects.

Path	Effect size	BOOTSE	BootLLCI	BootULCI
Total effect	0.23	0.047	0.136	0.323
Direct effect	0.172	0.048	0.078	0.266
Physical exercise → Satisfaction of exercise-related psychological needs → Subjective wellbeing	0.038	0.019	0.009	0.08
Physical exercise → Psychological resilience → Subjective wellbeing	0.015	0.009	0.006	0.036
Physical exercise → Satisfaction of exercise-related psychological needs → Psychological resilience → Subjective wellbeing	0.005	0.004	0.001	0.016

The total association between physical exercise and subjective wellbeing was 0.230 (95% CI [0.136, 0.323]), while the direct association remained significant (*β* = 0.172, 95% CI [0.078, 0.266]) after including the mediators, indicating a partial mediation pattern.

Three indirect pathways were observed. First, physical exercise was indirectly associated with subjective wellbeing through exercise psychological needs satisfaction (*β* = 0.038, 95% CI [0.009, 0.080]). Second, an indirect association was found through psychological resilience (*β* = 0.015, 95% CI [0.006, 0.036]). Third, a sequential indirect pathway was identified through exercise psychological needs satisfaction and psychological resilience (*β* = 0.005, 95% CI [0.001, 0.016]).

All confidence intervals excluded zero, suggesting that these indirect associations were statistically significant. The total indirect association was 0.058, accounting for 25.2% of the total association. Among the indirect pathways, the path through exercise psychological needs satisfaction showed the largest contribution.

## Discussion

This research paper focuses on the association between physical exercise and subjective wellbeing of Chinese students studying in South Korea and also focuses on the mediation process of psychological need satisfaction and psychological resiliency associated with exercise. The results provide empirical evidence regarding the mental health of international students and have potential theoretical and practical implications.

The findings suggest that subjective wellbeing is related to physical exercise with a significant positive correlation in Chinese students living in South Korea, as previous studies have also reported in general populations (Penedo and Dahn, 2005; Lin et al., 2022). Regular physical exercise may be associated with the secretion of neurotransmitters, endorphins, serotonin, and dopamine (Basso and Suzuki, 2017), which may in turn be linked to reductions in anxiety and depression and higher levels of subjective wellbeing. Physical exercise may also provide opportunities for social interaction and may help alleviate loneliness, which is highly significant to Chinese international students in foreign countries (Eather et al., 2023). An example is when playing team sports, students can socialize and work together by group activities, which may facilitate social bonding. These social experiences may be associated with higher life satisfaction and increased positive affect.

The connection between physical exercise and subjective wellbeing may be explained by psychological needs satisfaction, which is consistent with Self-Determination Theory (Ryan and Deci, 2017). Assuming that the three basic psychological needs (autonomy, competence, and relatedness) are satisfied in exercise contexts, students may experience more positive emotional states and higher life satisfaction (Zhang et al., 2022). In the present study, cross-cultural adaptation stress among international students was found to be lower among those who reported greater autonomy in exercise choices (Yin et al., 2024), suggesting that autonomy satisfaction may play an important role in its association with subjective wellbeing. More so, the sense of competence in exercise environments was more strongly associated with wellbeing, which is consistent with Chinese cultural values emphasizing capability development (Chen and Nakagawa, 2023). Relatedness needs were also associated with reduced loneliness in group sport settings, and this effect may be particularly relevant in the relatively individualistic social context of South Korea (Kim and Park, 2021).

Physical exercise and subjective wellbeing were also linked through psychological resilience, in line with Stress-Buffering Theory (Fletcher and Sarkar, 2016; Ong et al., 2010). Physical exercise may be associated with resilience through multiple pathways, such as neurobiological and behavioral processes, including the secretion of brain-derived neurotrophic factor (BDNF) and enhanced executive functioning (Hillman et al., 2008). Physical exercise may also be related to the development of self-efficacy (Bandura, 2018), while goal-setting and achievement processes may be associated with greater optimism (Seligman, 2006). This study also suggests that emotional regulation within psychological resilience was more strongly associated with subjective wellbeing, which may be related to Chinese cultural perspectives emphasizing emotional control (Liu, 2025). The proportion of the total association between physical exercise and subjective wellbeing explained through psychological resilience was approximately 42% (Kim and Park, 2021). This pattern may be more pronounced among female students, possibly due to greater adaptation-related stress (Lian, 2017).

The present findings extend previous research by identifying a chain-mediated pattern involving exercise-related psychological needs satisfaction and psychological resilience. Within the integrated framework of Self-Determination Theory and resilience theory (Ryan and Deci, 2017; Fletcher and Sarkar, 2016), satisfaction of psychological needs during exercise may be associated with higher levels of subjective wellbeing through its relationship with psychological resilience. The results indicate that psychological needs satisfaction was associated with subjective wellbeing both directly and indirectly through resilience (Lin, S., Li, L., Zheng, D., and Jiang, L., 2022). In line with neurophysiological perspectives, fulfillment of psychological needs in physical exercise may be related to psychological resilience through mechanisms such as regulation of the hypothalamic–pituitary–adrenal (HPA) axis (Kim and Park, 2021). In particular, relatedness satisfaction appeared to show a stronger association with resilience, which may reflect the importance of social support in collectivist cultural contexts (Chen and Nakagawa, 2023). This chain mediation pattern may be more evident in team sports settings, where participants experience both psychological needs satisfaction and enhanced resilience through group interaction (Kim and Park, 2021).

### Research limitations and future research prospects

This research has a number of limitations that need to be mentioned. First, the convenience sampling used in the selection of participants could have brought about sampling bias thus limiting the generalizability of the results. It is also recommended that further studies should use more rigorous sampling methods like stratified or random sampling to get a better representative sample and improve the external validity.

Second, data have been gathered using self-report tools, which are now prone to subjectivity and report bias on the part of respondents. This practice will create a possibility of having a greater social desirability bias and recall bias that might compromise the precision of the findings. To address future research, it might be proposed to use an objectively recorded method of data gathering, like a wearable motion detector or a digital activity tracker, to gather physical exercise data and minimise the possibility of measurement error.

Lastly, the current study is a cross-sectional research hence it is not in a position to provide causal relationships between physical exercise and psychological needs satisfaction and psychological resilience and subjective wellbeing. Despite the fact that significant associations have been identified, it was not possible to cause or infer. It is suggested that future longitudinal or experimental research should be conducted to evaluate how the physical exercise of international students, fulfillment of psychological needs, psychological resilience, and subjective wellbeing changes over the various stages they are in. These designs would offer more proof of causal relationships and would also prove more of the mechanisms behind these variables.

## Conclusion

The present study was grounded in self-determination theory and psychological resilience theory and aimed to examine the mechanisms through which physical exercise is associated with the subjective wellbeing of Chinese students studying in South Korea. The findings indicate that physical exercise was related to subjective wellbeing both directly and indirectly, with exercise-related psychological needs satisfaction and psychological resilience serving as key pathways.

These findings add to the existing literature by providing empirical evidence on the psychological benefits of physical exercise within a cross-cultural context. In particular, the study highlights the potential importance of motivational processes and resilience-related factors in understanding differences in wellbeing among international students.

Based on these findings, it may be useful for universities in South Korea to consider incorporating structured physical exercise programs for Chinese international students. Such programs could be designed to support the satisfaction of psychological needs, including autonomy, competence, and relatedness, while also being associated with the development of psychological resilience. By integrating these elements, universities may be better positioned to support the psychological wellbeing and adaptation of international students.

## Data Availability

The raw data supporting the conclusions of this article will be made available by the authors, without undue reservation.

## References

[ref1] Ávila M. P. W. Corrêa J. C. Lucchetti A. L. G. Lucchetti G. (2021). Relationship between mental health, resilience, and physical exercise in older adults: a 2-year longitudinal study. J. Aging Phys. Act. 30, 73–81. 34407504 10.1123/japa.2020-0264

[ref2] Bandhu D. Mohan M. M. Nittala N. A. P. Jadhav P. Bhadauria A. Saxena K. K. (2024). Theories of motivation: a comprehensive analysis of human behavior drivers. Acta Psychol. 244:104177. doi: 10.1016/j.actpsy.2024.104177, 38354564

[ref3] Bandura A. (1997). Self-Efficacy: The Exercise of Control. New York, NY: Freeman.

[ref4] Bandura A. (2018). Toward a psychology of human agency: pathways and reflections. Perspect. Psychol. Sci. 13, 130–136. doi: 10.1177/1745691617699280, 29592657

[ref5] Basso J. C. Suzuki W. A. (2017). The effects of acute exercise on mood, cognition, neurophysiology, and neurochemical pathways: a review. Brain Plasticity 2, 127–152. doi: 10.3233/BPL-160040, 29765853 PMC5928534

[ref6] Blustein D. L. Lysova E. I. Duffy R. D. (2023). Understanding decent work and meaningful work. Annu. Rev. Organ. Psychol. Organ. Behav. 10, 289–314. doi: 10.1146/annurev-orgpsych-031921-024847

[ref7] Brunsting N. C. Zachry C. Takeuchi R. (2018). Predictors of undergraduate international student psychosocial adjustment to US universities: a systematic review from 2009-2018. Int. J. Intercult. Relat. 66, 22–33. doi: 10.1016/j.ijintrel.2018.06.002

[ref9001] Campbell A. Converse P. E. Rodgers W. L. (1976). The Quality of American Life: Perceptions, Evaluations, and Satisfactions. New York, NY: Russell Sage Foundation. doi: 10.2307/2148954

[ref8] Chen C. Nakagawa S. (2023). Physical exercise for cognitive health promotion: an overview of the underlying neurobiological mechanisms. Ageing Res. Rev. 86:101868. doi: 10.1016/j.arr.2023.101868, 36736379

[ref9] Cosco T. D. Kaushal A. Hardy R. Richards M. Kuh D. Stafford M. (2017). Operationalising resilience in longitudinal studies: a systematic review of methodological approaches. J. Epidemiol. Community Health 71, 98–104. doi: 10.1136/jech-2015-206980, 27502781 PMC5256275

[ref10] De Nys L. Anderson K. Ofosu E. F. Ryde G. C. Connelly J. Whittaker A. C. (2022). The effects of physical exercise on cortisol and sleep: a systematic review and meta-analysis. Psychoneuroendocrinology 143:105843. doi: 10.1016/j.psyneuen.2022.105843, 35777076

[ref12] Eather N. Wade L. Pankowiak A. Eime R. (2023). The impact of sports participation on mental health and social outcomes in adults: a systematic review and the ‘mental health through sport’ conceptual model. Syst. Rev. 12:102. doi: 10.1186/s13643-023-02264-8, 37344901 PMC10286465

[ref13] Fletcher D. Sarkar M. (2016). Mental fortitude training: an evidence-based approach to developing psychological resilience for sustained success. J. Sport Psychol. Action 7, 135–157. doi: 10.1080/21520704.2016.1255496

[ref14] Göttgens I. Oertelt-Prigione S. (2021). The application of human-centered design approaches in health research and innovation: a narrative review of current practices. JMIR Mhealth Uhealth 9:e28102. doi: 10.2196/28102, 34874893 PMC8691403

[ref15] Hascher T. Waber J. (2021). Teacher well-being: a systematic review of the research literature from the year 2000–2019. Educ. Res. Rev. 34:100411. doi: 10.1016/j.edurev.2021.100411

[ref16] Heng T. T. (2018). Different is not deficient: contradicting stereotypes of Chinese international students in US higher education. Stud. High. Educ. 43, 22–36. doi: 10.1080/03075079.2016.1152466

[ref17] Hilber J (2024). The Power of Resilience: Understanding Student Well-Being Through Self Determination Theory [Doctoral Dissertation]

[ref18] Hillman C. H. Erickson K. I. Kramer A. F. (2008). Be smart, exercise your heart: exercise effects on brain and cognition. Nat. Rev. Neurosci. 9, 58–65. doi: 10.1038/nrn2298 18094706

[ref19] Jackson S. B. Stevenson K. T. Larson L. R. Peterson M. N. Seekamp E. (2021). Outdoor activity participation improves adolescents’ mental health and well-being during the COVID-19 pandemic. Int. J. Environ. Res. Public Health 18:2506. doi: 10.3390/ijerph18052506, 33802521 PMC7967628

[ref20] Khaldi A. Bouzidi R. Nader F. (2023). Gamification of e-learning in higher education: a systematic literature review. Smart Learn. Environ. 10:10. doi: 10.1186/s40561-023-00227-z, 40478066 PMC9887250

[ref21] Kim Y. Kuan G. (2022). A cross-cultural comparison of college students’ physical exercise in Korea and Malaysia using the transtheoretical model. Int. J. Sport Psychol. 53, 1–15.

[ref22] Kim H. Park J. (2021). Social connectedness in team sports and mental health among international students: a cross-cultural comparison. Int. J. Sport Psychol. 52, 245–260.

[ref23] Kristiana I. F. Karyanta N. A. Simanjuntak E. Prihatsanti U. Ingarianti T. M. Shohib M. (2022). Social support and acculturative stress of international students. Int. J. Environ. Res. Public Health 19:6568. doi: 10.3390/ijerph19116568, 35682152 PMC9180523

[ref24] Lee C. Kim S. Owens M. Liechty T. Kim J. (2019). Engaging with sports related serious leisure and acculturation among Korean graduate students. Ann. Leisure Res. 22, 247–263. doi: 10.1080/11745398.2018.1496463

[ref25] Lee A. R. Lee H. K. (2020). The effects of acculturative stress, career stress, and social support on depression in Korean international students in China. J. Korean Acad. Community Health Nurs. 31, 96–106. doi: 10.12799/jkachn.2020.31.1.96

[ref26] Li L. Wang P. Zhao Q. Liu Z. Li S. Wang X. (2024). Latent profile analysis of depressive symptoms in college students and its relationship with physical exercise. J. Affect. Disord. 350, 95–103. 10.1016/j.jad.2024.01.214 38296059

[ref27] Lian Z. (2017). Predictors of Depression/Anxiety, Mental Health Service Utilization, and Help-Seeking for Chinese International Students: Role of Acculturation, Microaggressions, Social Support, Coping Self-Efficacy, Stigma, and College Staffs' Cultural Competence and Cultural Humility [Doctoral Dissertation]. Teachers College, Columbia University. New York, NY: Guilford Press.

[ref28] Liang D. Q. (1994). Stress levels among college students and their relationship with physical exercise. Chin. Ment. Health J. 8, 5–6.

[ref29] Lin S. Li L. Zheng D. Jiang L. (2022). Physical exercise and undergraduate students’ subjective well-being: mediating roles of basic psychological need satisfaction and sleep quality. Behavi. Sci. 12:316. doi: 10.3390/bs12090316, 36135120 PMC9495405

[ref30] Liu P. (2025). Stress buffering effects of physical exercise in adolescents: the moderating role of physical exercise attitudes. BMC Public Health 25:463. doi: 10.1186/s12889-025-21674-y, 39910517 PMC11800644

[ref31] Mandolesi L. Polverino A. Montuori S. Foti F. Ferraioli G. Sorrentino P. . (2018). Effects of physical exercise on cognitive functioning and wellbeing: biological and psychological benefits. Front. Psychol. 9:347071. doi: 10.3389/fpsyg.2018.00509, 29755380 PMC5934999

[ref32] Martín-Rodríguez A. Gostian-Ropotin L. A. Beltrán-Velasco A. I. Belando-Pedreño N. Simón J. A. López-Mora C. . (2024). Sporting mind: the interplay of physical activity and psychological health. Sports 12:37. doi: 10.3390/sports12010037, 38275986 PMC10819297

[ref33] Ministry of Culture, Sports and Tourism, Republic of Korea. (2022). Enforcement decree of the National Sports Promotion Act. Available online at: https://www.mcst.go.kr

[ref34] Ministry of Education. (2020) Study in China program. Available online at: http://www.moe.gov.cn

[ref35] Ministry of Education, Republic of Korea. (2021). Support program for foreign students. Available online at: https://www.moe.go.kr

[ref36] Ng J. Y. Lonsdale C. Hodge K. (2011). The basic needs satisfaction in sport scale (BNSSS): instrument development and initial validity evidence. Psychol. Sport Exerc. 12, 257–264. doi: 10.1016/j.psychsport.2010.10.006

[ref9002] Nunnally J. C. Bernstein I. H. (1994). Psychometric Theory. 3rd Edn. New York, NY: McGraw-Hill., 16639173

[ref37] Ong A. D. Bergeman C. S. Chow S. M. (2010). “Positive emotions as a basic building block of resilience in adulthood,” in Handbook of Adult Resilience, (), 81–93.

[ref38] Penedo F. J. Dahn J. R. (2005). Exercise and well-being: a review of mental and physical health benefits associated with physical activity. Curr. Opin. Psychiatry 18, 189–193. doi: 10.1097/00001504-200503000-00013, 16639173

[ref39] Ryan R. M. Deci E. L. (2017). Self-Determination Theory: Basic Psychological Needs in Motivation, Development, and Wellness. New York, NY, USA: Columbia University.

[ref40] Seligman M. E. (2006). Learned Optimism: How to change your mind and your life. New York, NY: Vintage.

[ref41] Smith P. B. Khawaja N. G. (2011). A review of the acculturation experiences of international students. Int. J. Intercult. Relat. 35, 699–713. doi: 10.1016/j.ijintrel.2011.08.004

[ref42] Song J. Liu Z. Z. Huang J. Wu J. S. Tao J. (2021). Effects of aerobic exercise, traditional Chinese exercises, and meditation on depressive symptoms of college student: a meta-analysis of randomized controlled trials. Medicine 100:e23819. doi: 10.1097/MD.0000000000023819, 33429742 PMC7793414

[ref43] State Council. (2016). Outline of the “Healthy China 2030” plan. Available online at: http://www.gov.cn

[ref44] Toth E. E. Ihász F. Ruíz-Barquín R. Szabo A. (2023). Physical exercise and psychological resilience in older adults: a systematic review of the literature. J. Aging Phys. Act. 32, 276–286. 37699587 10.1123/japa.2022-0427

[ref45] Van Agteren J. Iasiello M. Lo L. Bartholomaeus J. Kopsaftis Z. Carey M. . (2021). A systematic review and meta-analysis of psychological interventions to improve mental wellbeing. Nat. Hum. Behav. 5, 631–652. doi: 10.1038/s41562-021-01093-w, 33875837

[ref46] Wang J. Xu X. Wu Q. Zhou C. Yang G. (2024). The mediating effect of subject well-being between physical activity and the internet addiction of college students in China during the COVID-19 pandemic: a cross-sectional study. Front. Public Health 12:1368199. doi: 10.3389/fpubh.2024.1368199, 38645442 PMC11026853

[ref47] Wilczewski M. Alon I. (2023). Language and communication in international students’ adaptation: a bibliometric and content analysis review. High. Educ. 85, 1235–1256. doi: 10.1007/s10734-022-00888-8, 35855684 PMC9274966

[ref48] Yang M (2019). Motivation toward physical education and its relation to academic stress among Chinese adolescent: a cultural perspective on self-determination theory

[ref49] Yim H. Kim A. C. H. Du J. James J. D. (2023). Sport participation, acculturative stress, and depressive symptoms among international college students in the United States. Front. Psych. 14:1104325. doi: 10.3389/fpsyt.2023.1104325, 36937740 PMC10017837

[ref50] Yin C. Kim S. H. Kim Y. H. (2024). Factors associated with changes in physical exercise levels among Chinese international students in South Korea. Phys. Act. Nutr. 28, 031–041. doi: 10.20463/pan.2024.0030, 39934628 PMC11811621

[ref51] Yu X. N. Zhang J. X. (2007). Comparative application of the self-resilience scale and Connor-Davidson resilience scale. Psychol. Sci. 30, 1169–1171.

[ref52] Zerengok D. Guzel P. Ozbey S. (2018). The impact of leisure participation on social adaptation of international students. J. Educ. Train. Stud. 6, 1–9. doi: 10.11114/jets.v6i2.2680

[ref53] Zhang J. Goodson P. (2011). Acculturation and psychosocial adjustment of Chinese international students: examining mediation and moderation effects. Int. J. Intercult. Relat. 35, 614–627. doi: 10.1016/j.ijintrel.2010.11.004

[ref54] Zhang Y. Ren M. Zou S. (2022). Effect of physical exercise on college students’ life satisfaction: mediating role of competence and relatedness needs. Front. Psychol. 13:930253. doi: 10.3389/fpsyg.2022.930253, 35967665 PMC9372330

[ref55] Zhu J. (2014). The Relationship between Perceived Autonomous Support from Significant Others and Adolescents' Exercise Behavior: An Approach based on Self-Determination Theory [Master's Thesis]. Beijing, China: Capital University of Physical Education and Sport.

